# Effect of renal denervation on glucose metabolism in hypertensive patients with and without chronic kidney disease

**DOI:** 10.1093/ckj/sfaf184

**Published:** 2025-06-18

**Authors:** Venera Bytyqi, Dennis Kannenkeril, Axel Schmid, Kristina Striepe, Agnes Bosch, Marina V Karg, Mario Schiffer, Michael Uder, Roland E Schmieder

**Affiliations:** Department of Nephrology and Hypertension, University Hospital Erlangen, Friedrich-Alexander University Erlangen-Nuremberg, Erlangen, Germany; Department of Nephrology and Hypertension, University Hospital Erlangen, Friedrich-Alexander University Erlangen-Nuremberg, Erlangen, Germany; Institute of Radiology, University Hospital Erlangen, Friedrich-Alexander University Erlangen-Nuremberg, Erlangen, Germany; Department of Nephrology and Hypertension, University Hospital Erlangen, Friedrich-Alexander University Erlangen-Nuremberg, Erlangen, Germany; Department of Nephrology and Hypertension, University Hospital Erlangen, Friedrich-Alexander University Erlangen-Nuremberg, Erlangen, Germany; Department of Nephrology and Hypertension, University Hospital Erlangen, Friedrich-Alexander University Erlangen-Nuremberg, Erlangen, Germany; Department of Nephrology and Hypertension, University Hospital Erlangen, Friedrich-Alexander University Erlangen-Nuremberg, Erlangen, Germany; Institute of Radiology, University Hospital Erlangen, Friedrich-Alexander University Erlangen-Nuremberg, Erlangen, Germany; Department of Nephrology and Hypertension, University Hospital Erlangen, Friedrich-Alexander University Erlangen-Nuremberg, Erlangen, Germany

**Keywords:** chronic kidney disease, glucose metabolism, renal denervation

## Abstract

**Backgroun:**

Sympathetic overactivation is associated with numerous pathologies, including arterial hypertension, diabetes, metabolic syndrome and chronic kidney disease (CKD). Renal denervation (RDN) has emerged as an adjacent therapy for the management of hypertension. By modulating sympathetic activity in the whole body, RDN has shown conflicting results regarding insulin secretion and glucose homeostasis. The aim of this study is to analyse the impact of RDN on glycaemic control in patients with CKD.

**Methods:**

A total of 155 hypertensive patients with (*n* = 45) or without CKD (*n* = 110) were included in this post hoc analysis. All patients underwent radiofrequency-, ultrasound- or alcohol injection–based RDN. Fasting plasma glucose (FPG) and haemoglobin A1c levels were measured at baseline, 3 months and 6 months after RDN in parallel with the office and 24-h ambulatory blood pressure. CKD was defined either by clinical diagnosis, an estimated glomerular filtration rate (eGFR) of 15–59 ml/min/1.73 m^2^ and/or A2/A3 albuminuria in hypertensive patients, repeatedly confirmed, or several of these criteria.

**Results:**

In the total study cohort, FPG decreased by 5.1 ± 29.1 mg/dl (*P* = .032) and by 7.9 ± 32.7 mg/dl (*P* = .003) at 3 and 6 months after RDN, respectively. The change in FPG levels was related to the change in 24-h systolic BP (*r* = 0.286, *P* = .008) 3 months after RDN. Among patients with CKD, FPG levels decreased by 13.5 ± 43.5 mg/dl at 3 months (*P* = .043) and by 17.1 ± 45.2 mg/dl at 6 months (*P* = .015) following RDN. These reductions were greater compared with the non-CKD group (*P* = .021 and *P* = .024, respectively). After excluding patients on oral antidiabetic or insulin therapy, patients with CKD (but not those without CKD) exhibited a reduction in FPG levels of 6.7 ± 15.3 mg/dl (*P* = .043) at 6 months post-RDN. No significant changes were observed in eGFR in either group.

**Conclusion:**

We observed that FPG levels improved to a greater extent in hypertensive patients with CKD after RDN. Thus RDN may have a broader therapeutic impact beyond blood pressure reduction in CKD patients.

## Graphical Abstract

**Graphical Abstract:**
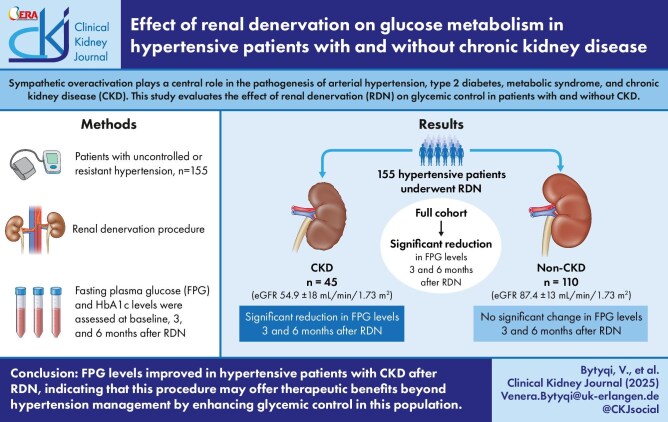

KEY LEARNING POINTS **What was known:**

- Sympathetic overactivation is associated with various chronic conditions, including arterial hypertension, diabetes, metabolic syndrome and chronic kidney disease (CKD).
- Renal denervation (RDN) has been primarily developed as an adjunctive therapy for managing arterial hypertension but has shown conflicting results in its impact on insulin secretion and glucose homeostasis.

**This study adds:**

- RDN significantly improves fasting plasma glucose levels in hypertensive patients with CKD, irrespective of antidiabetic or insulin therapy. No comparable improvements were observed in hypertensive patients without CKD.
- RDN significantly reduces office and 24-h ambulatory blood pressure in hypertensive patients, regardless of CKD status.

**Potential impact:**

- Our findings suggest that RDN might have an additional benefit for hypertensive patients with CKD, addressing both blood pressure and glycaemic control, thereby broadening its therapeutic potential.

## INTRODUCTION

The sympathetic nervous system (SNS) plays a pivotal role in the regulation of cardiovascular and metabolic functions, maintaining critical physiological processes such as heart rate, blood pressure (BP), vascular tone, glucose metabolism, lipid utilization and energy balance [1–3]. While its activation is essential for preserving homeostasis under normal conditions, chronic overactivation of the SNS has been identified as a key pathophysiological mechanism contributing to a range of cardiometabolic disorders, including arterial hypertension, diabetes mellitus, metabolic syndrome and chronic kidney disease (CKD) [1, 4–6].

In particular, increased renal sympathetic drive is commonly observed in patients with hypertension and is strongly associated with metabolic syndrome components. Furthermore, studies highlight a reciprocal interaction where sympathetic overactivity exacerbates insulin resistance while hyperinsulinemia further stimulates sympathetic activation, creating a self-perpetuating cycle that worsens both metabolic and cardiovascular dysfunction [7–10].

A deeper understanding of the SNS's involvement in these pathophysiological processes has led to the development of innovative therapeutic approaches aimed at restoring physiological balance and improving clinical outcomes [6, 11]. Catheter-based renal denervation (RDN) has predominantly emerged as an adjacent therapy for the management of hypertension. The underlying rationale of RDN is to interrupt the nerve traffic of both afferent sensory and efferent sympathetic nerves located in the perivascular space surrounding the renal arteries. By reducing sympathetic influence on renal vascular resistance, renin release and sodium reabsorption, RDN targets key factors that contribute to the maintenance and progression of hypertension [12]. Over the years, numerous randomized, sham-controlled trials have demonstrated the safety and efficacy of RDN in both short- and long-term follow-up studies [13–15]. Given the established link between sympathetic activity and metabolic dysfunction in clinical studies, RDN has shown conflicting results regarding insulin secretion and glucose homeostasis [16–19]. The aim of this study was to analyse the impact of RDN on glycaemic control in patients with or without CKD.

## MATERIALS AND METHODS

### Study design

This is a post hoc analysis of 155 hypertensive patients who underwent RDN. Of these, 98 patients were part of the Renal Denervation in Treatment-Resistant Hypertension trial (NCT01687725), an investigator-initiated study performed only in our Erlanger centre. The remaining 57 patients were involved in various clinical trials, either randomized or non-randomized and sham controlled or non-sham controlled (NCT02439749, NCT02649426, NCT02570113, NCT02649426, NCT02439775, NCT04311086, NCT03614260, NCT03503773). The studies were conducted at the Clinical Research Center of the Department of Nephrology and Hypertension at University Hospital Erlangen-Nuremberg, Germany. All study protocols received approval from the local ethical review committee (University of Erlangen-Nuremberg) and adhered to the Declaration of Helsinki guidelines. Written informed consent was obtained from all participants before their inclusion in the study.

### Study cohort

The study population consisted of adult patients with uncontrolled hypertension, including patients with treatment-resistant hypertension taking at least three antihypertensive drugs (including one diuretic), patients with one to three antihypertensive drugs and patients without any antihypertensive medication. Key exclusion criteria were secondary causes of arterial hypertension, pregnancy, type 1 diabetes, significant renal artery abnormalities, previous renal RDN and contraindications for undergoing the RDN procedure. Despite slight variations in BP criteria across different studies, all cases of uncontrolled hypertension were confirmed using 24-h ambulatory blood pressure (ABP) monitoring, thereby ruling out white-coat hypertension. Furthermore, standard care procedures included screening for secondary hypertension causes in all patients [20].

### RDN procedure

The RDN procedure was performed with four different denervation catheters: a radiofrequency-based Symplicity-Flex catheter (Ardian, Palo Alto, CA, USA), a radiofrequency-based Symplicity-Spyral catheter (Medtronic, Santa Rosa, CA, USA), an ultrasound-based Paradise catheter (ReCor Medical, Palo Alto, CA, USA) and an alcohol infusion–based Peregrine system catheter (Ablative Solutions, Kalamazoo, MI, USA). All procedures were performed through femoral access, treating both renal arteries in a single session. A detailed description of the procedure is available in previously published studies [21–25].

### Assessments

The baseline assessments included physical examinations, measurements of office and 24-h ABP, the collection of medical history and antihypertensive medication data and blood and urine tests. Fasting plasma glucose (FPG) and glycated haemoglobin (HbA1c) concentrations were measured at baseline, 3 months and 6 months after the RDN procedure.

### Statistical analysis

All statistical analyses were performed with SPSS Statistics 28.0 (IBM, Armonk, NY, USA) and normally distributed data are expressed as mean ± standard deviation (SD). Changes in FPG, HbA1c and office and 24-h BPs were analysed from baseline to 3 and 6 months with a paired samples *t*-test. The unpaired *t*-test was applied to compare continuous variables and the chi-squared test was performed to compare categorical variables between the groups. A two-tailed *P*-value <.05 was considered statistically significant. Bivariate correlation analyses, using Pearson's test, were used to evaluate the relationship between the reduction in 24-h systolic BP and the reduction in FPG levels after RDN.

## RESULTS

### Baseline characteristics

The baseline characteristics of the study cohort, as detailed in Table 1, include 155 patients diagnosed with uncontrolled hypertension, including resistant hypertension. The age of participants ranged from 33 to 79 years, with a mean age of 59 years. The cohort was predominantly male, accounting for 77% of the total population. There were no significant differences in demographic data between patients with or without CKD. However, comorbidities were more prevalent in the CKD group, with 57% of patients diagnosed with type 2 diabetes, 53% presenting with hyperlipidaemia and 38% having a history of coronary heart disease.

**Table 1:** Clinical characteristics of the study cohort at the baseline.

Clinical characteristics	All (*N* = 155)	CKD (*n* = 45)	Non-CKD (*n* = 110)	*P*-value
Demographic data				
Age (years)	59.1 ± 10.1	61.4 ± 11.3	58.2 ± 9.5	.070
Gender (male/female)	119/36	36/9	83/27	.676
Weight (kg)	91.5 ± 16.9	90.2 ± 18.6	92.0 ± 16.2	.566
Body mass index (kg/m^2^)	30.0 ± 4.7	30.0 ± 4.9	29.9 ± 4.6	.458
Comorbidities, *n* (%)				
Type 2 diabetes	49 (32)	26 (57)	23 (21)	**.001**
Coronary artery disease	32 (21)	17 (38)	15 (14)	**.002**
Left ventricular hypertrophy	23 (15)	13 (29)	10 (9)	**.003**
Hyperlipidaemia	57 (37)	24 (53)	33 (30)	**.010**
History of stroke/TIA	16 (10)	9 (20)	7 (6)	**.018**
CKD	45 (29)	45 (100)	0 (0)	**<.001**
Current smoker	14 (9)	2 (4)	12 (11)	.167
Office BP				
Systolic (mmHg)	156.4 ± 18.8	153.7 ± 19.8	157.6 ± 18.3	.244
Diastolic (mmHg)	89.4 ± 13.1	84.4 ± 13.4	91.4 ± 12.5	**.002**
Heart rate (bpm)	68.5 ± 12.7	68.5 ± 14.4	68.6 ± 12.0	.960
24-h ABP				
Systolic (mmHg)	146.0 ± 12.0	145.1 ± 11.2	145.6 ± 12.4	.623
Diastolic (mmHg)	88.6 ± 10.9	86.0 ± 8.1	91.3 ± 10.7	.051
Heart rate (bpm)	68.0 ± 10.3	70.2 ± 9.1	68.7 ± 10.6	.581
Laboratory values				
HbA1c (%)	6.1 ± 0.9	6.3 ± 0.8	6.0 ± 1.0	.086
FPG (mg/dl)	119.9 ± 49.3	140.7 ± 66.1	111.4 ± 37.7	**<.001**
Serum creatinine (mg/ml)	1.0 ± 0.4	1.4 ± 0.4	0.9 ± 0.2	**<.001**
eGFR (ml/min/1.73 m^2^)	78.6 ± 21.0	54.9 ± 18.7	87.4 ± 13.8	**<.001**
Serum triglyceride (mg/dl)	173.4 ± 98.6	175.9 ± 83.4	172.2 ± 105.5	.835
Serum cholesterol (mg/dl)	207.0 ± 47.2	185.9 ± 45.4	217.1 ± 44.9	**<.001**
Serum LDL cholesterol (mg/dl)	138.8 ± 37.5	123.4 ± 37.4	146.2 ± 35.5	**<.001**
Serum HDL cholesterol (mg/dl)	49.5 ± 14.5	45.3 ± 11.1	51.5 ± 15.5	**.019**
Haemoglobin (g/dl)	14.3 ± 1.4	13.6 ± 1.5	14.5 ± 1.2	**<.001**
Haematocrit (%)	41.9 ± 4.0	40.2 ± 4.6	42.7 ± 3.5	**<.001**
Medication				
Antihypertensive drugs (*n*)	4.41 ± 2.5	5.7 ± 2.1	3.9 ± 2.5	**<.001**
Oral antidiabetic, *n* (%)	21 (14)	9 (20)	12 (11)	.249
Insulin therapy, *n* (%)	12 (12)	10 (26)	2 (2)	**.001**
Oral antidiabetic and insulin therapy, *n* (%)	7 (5)	2 (4)	5 (6)	.978

Data are presented as mean ± SD unless stated otherwise.

Significant values in bold.

TIA: transient ischaemic attack; LDL: low-density lipoprotein; HDL: high-density lipoprotein.

### FPG and HbA1c levels in the entire cohort

**Figure 1:**
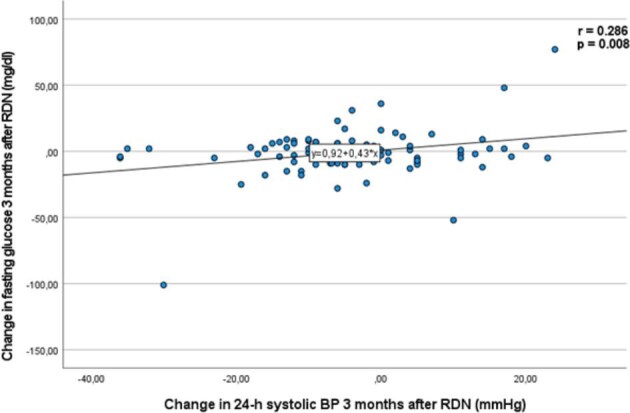
Correlation of the change in FPG and the change in 24-h ambulatory systolic BP 3 months after RDN. Data are presented as mean ± SD.

We observed a significant reduction in FPG levels in the whole cohort population 3 and 6 months after the procedure. FPG decreased by 5.1 ± 29.1 mg/dl (*P* = .032) and by 7.9 ± 32.7 mg/dl (*P* = .003) at 3 and 6 months after RDN, respectively. The change in FPG levels correlated with the change in 24-h systolic BP (r = 0.286, *P* = .008) 3 months after RDN in the whole cohort (Fig. 1). No significant changes regarding HbA1c were observed (Table 2B).

**Table 2:** Changes in FPG and HbA1c levels 3 and 6 months after RDN.

Group A	FPG at baseline (mg/dl)	FPG 3 months after RDN (mg/dl)	*P*-value (baseline versus 3 months after RDN)	FPG 6 months after RDN (mg/dl)	*P*-value (baseline versus 6 months after RDN)
All (*N* = 155)	119.9 ± 49.3	114.8 ± 41.6	**0.032**	112.1 ± 32.1	**.003**
CKD (*n* = 45)	140.7 ± 66.1	127.2 ± 44.9	**0.043**	123.6 ± 36.6	**.015**
Non-CKD (*n* = 110)	111.4 ± 37.7	109.8 ± 39.2	0.395	107.3 ± 28.9	.092
Group B	HbA1c at baseline (%)	HbA1c 3 months after RDN (%)	*P*-value (baseline versus 3 months after RDN)	HbA1c 6 months after RDN (%)	*P*-value (baseline versus 6 months after RDN)
All (*N* = 89)	6.14 ± 1.0	6.10 ± 0.9	0.360	6.08 ± 1.0	.214
CKD (*n* = 27)	6.29 ± 0.8	6.36 ± 0.8	0.194	6.29 ± 0.9	.962
Non-CKD (*n* = 62)	6.08 ± 1.0	5.99 ± 0.9	0.120	5.99 ± 1.0	.165

Data are presented as mean ± SD.

Significant values in bold.

To rule out any potential effects of concurrent antidiabetic therapy, including insulin therapy, we performed a subgroup analysis excluding 40 patients undergoing such treatments. In this subset, we observed a reduction in FPG levels (*P* = .030) (Table 3A) and HbA1c levels (*P* = .017) 6 months following RDN.

**Table 3:** Changes in FPG and HbA1c levels 3 and 6 months after RDN, excluding the patients receiving insulin or oral antidiabetic therapy or both antidiabetic therapies.

Group A	FPG at baseline (mg/dl)	FPG 3 months after RDN (mg/dl)	*P*-value (baseline versus 3 months after RDN)	FPG 6 months after RDN (mg/dl)	*P*-value (baseline versus 6 months after RDN)
All (*N* = 115)	102.5 ± 17.5	101.0 ± 19.4	0.255	99.8 ± 14.0	**.030**
CKD (*n* = 24)	108.3 ± 19.1	103.5 ± 16.3	0.053	101.6 ± 11.3	**.043**
Non-CKD (*n* = 91)	101.0 ± 16.8	100.4 ± 20.2	0.690	99.3 ± 14.6	.210
Group B	HbA1c at baseline (%)	HbA1c 3 months after RDN (%)	*P*-value (baseline versus 3 months after RDN)	HbA1c 6 months after RDN (%)	*P*-value (baseline versus 6 months after RDN)
All (*N* = 67)	5.79 ± 0.6	5.76 ± 0.5	0.538	5.67 ± 0.4	**.017**
CKD (*n* = 18)	5.99 ± 0.4	6.07 ± 0.5	0.195	5.90 ± 0.5	.188
Non-CKD (*n* = 49)	5.72 ± 0.6	5.66 ± 0.4	0.259	5.59 ± 0.4	**.043**

Data are presented as mean ± SD.

Significant values in bold.

### Subgroup analysis—CKD

At baseline, the mean estimated glomerular filtration rate (eGFR) was 54.9 ± 18 ml/min/1.73 m^2^ in the CKD group and 87.4 ± 13 ml/min/1.73 m^2^ in the non-CKD group. No significant change in eGFR was observed in patients with or without CKD at 3 (56.0 ± 22 ml/min/1.73 m^2^ and 86.7 ± 13 ml/min/1.73 m^2^, respectively) and 6 months (52.0 ± 22 ml/min/1.73 m^2^ and 86.7 ± 14 ml/min/1.73 m^2^, respectively) after RDN.

**Figure 2:**
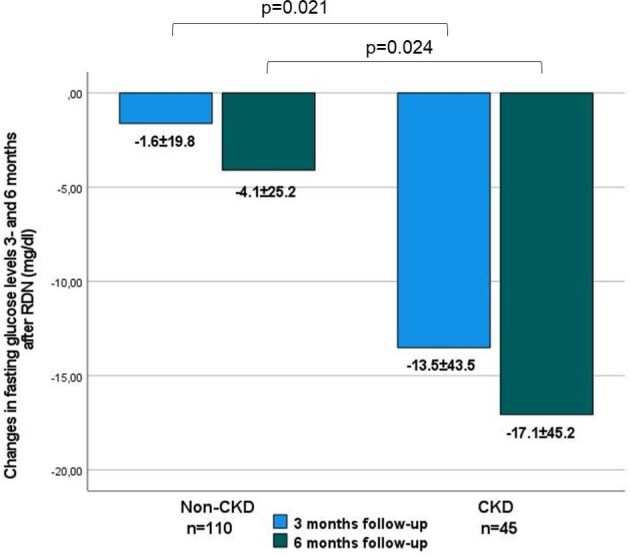
Changes in FPG levels 3 and 6 months after RDN in the CKD and non-CKD subgroups. Data are presented as mean ± SD.

In patients with CKD, mean FPG levels decreased by 13.4 ± 43.5 mg/dl at 3 months (*P* = .043) and by 17.1 ± 45.2 mg/dl at 6 months (*P* = .015) after RDN (Table 2). In patients without CKD, we observed no significant reductions in FPG levels. The reductions in FPG observed at 3 and 6 months post-RDN were greater in the CKD group compared with the non-CKD group (*P* = .021 and .024, respectively) (see Fig. 2).

Consistent with the entire cohort, after excluding patients on oral antidiabetic or insulin therapy, patients with CKD (but not those without CKD) exhibited a reduction in FPG levels by 6.7 mg/dl (*P* = .043) at 6 months post-RDN. Changes in HbA1c did not reach statistical significance in patients with CKD, potentially related to the faster turnover of erythrocytes in CKD, whereas in patients without CKD, HbA1c was reduced 6 months post-RDN (*P* = .043).

### Systolic and diastolic ABP reduction

In the entire cohort, office and 24-h BP decreased from baseline at all time points following RDN (Table 4A, *P* < .001). At 6 months post-RDN, office BP decreased by 13.6 ± 18.7/5.8 ± 10.6 mmHg (*P* < .001) and 24-h ABP by 6.0 ± 14.8/4.0 ± 8.6 mmHg (*P* < .001).

**Table 4:** Office and 24-h systolic and diastolic BP at baseline and changes 3 and 6 months after RDN in the **(A)** whole cohort and **(B)** CKD and non-CKD subgroups.

All patients (n = 155)										
**B:**BP	Baseline		3 months after RDN		6 months after RDN		*P*-value (baseline versus 3 months)		*P*-value (baseline versus 6 months)	
Office systolic (mmHg)	156.4 ± 18.8		148.6 ± 19.4		142.8 ± 19.0		**<.001**		**<.001**	
Office diastolic (mmHg)	89.4 ± 13.1		85.5 ± 12.9		82.7 ± 11.2		**<.001**		**<.001**	
Office heart rate (bpm)	68.5 ± 12.7		66.4 ± 11.9		66.9 ± 11.3		**<.001**		**.039**	
24-h systolic (mmHg)	146.0 ± 12.0		141.9 ± 14.3		139.9 ± 14.7		**.005**		**<.001**	
24-h diastolic (mmHg)	89.8 ± 10.2		87.6 ± 10.4		85.8 ± 9.3		**.008**		**<.001**	
24-h heart rate (bpm)	69.0 ± 10.2		68.1 ± 9.6		69.0 ± 9.9		.174		.968	
**B:**	CKD (*n* = 45)					Non-CKD (*n* = 110)				
BP	Baseline	3 months after RDN	6 months after RDN	*P*-value (baseline versus 3 months)	*P*-value (baseline versus 6 months)	Baseline	3 months after RDN	6 months after RDN	*P*-value (baseline versus 3 months)	*P*-value (baseline versus 6 months)
Office systolic (mmHg)	153.7 ± 19.8	148.8 ± 20.5	141.9 ± 20.1	.102	**.001**	157.6 ± 18.3	148.5 ± 19.0	143.2 ± 18.8	**<.001**	**<.001**
Office diastolic (mmHg)	84.4 ± 13.4	81.1 ± 12.1	78.3 ± 11.5	.054	**.001**	91.4 ± 12.5	87.3 ± 12.8	84.5 ± 10.7	**<.001**	**<.001**
Office heart rate (bpm)	68.5 ± 14.4	66.7 ± 13.4	65.2 ± 11.4	.143	**.038**	68.6 ± 12.0	66.3 ± 11.3	67.6 ± 11.3	**.003**	.278
24-h systolic (mmHg)	145.1 ± 11.2	140.1 ± 11.5	140.5 ± 10.7	**.032**	.087	146.6 ± 12.4	143.0 ± 14.5	139.3 ± 14.3	**.031**	**<.001**
24-h diastolic (mmHg)	86.0 ± 8.1	83.6 ± 9.0	83.6 ± 9.8	.081	.149	91.3 ± 10.7	89.3 ± 10.4	86.7 ± 9.0	.066	**<.001**
24-h heart rate (bpm)	70.2 ± 9.0	67.7 ± 8.0	67.7 ± 8.6	.118	.060	68.7 ± 10.6	68.2 ± 10.1	69.4 ± 10.4	.547	.299

Data are presented as mean ± SD.

Significant values in bold.

In the CKD group at 6 months post-RDN, office BP decreased by 11.8 ± 22.5/6.0 ± 11.7 mmHg (*P* = .001) and 24-h BP decreased by 4.5 ± 10.9/2.4 ± 7.0 mmHg (*P* = .087), whereas in the non-CKD group office BP decreased by 14.4 ± 16.9/6.9 ± 10.5 mmHg (*P* < .001) and 24-h BP by 7.3 ± 13.8/4.6 ± 8.9 mmHg (*P* < .001), without any significant difference between the two groups.

## DISCUSSION

In this study, we aimed to analyse the impact of RDN on glycaemic control in hypertensive patients with or without CKD after RDN. Our findings show a significant reduction in FPG levels in the whole study cohort 3 and 6 months following RDN. This reduction aligns with the work of Mahfoud *et al.*, who reported that RDN not only lowers BP but also improves glucose metabolism in patients with resistant hypertension [16]. Accordingly, inhibition of the SNS by moxonidine has been shown to enhance glucose metabolism by reducing glucagon secretion, increasing blood flow to skeletal muscles and decreasing glycogenolysis and gluconeogenesis [26].

In the CKD patients, FPG levels were significantly reduced after RDN and showed a progressive decrease over time after RDN. No significant reduction in FPG concentrations was observed in the non-CKD group. Since a few patients were on antidiabetic therapies, including insulin therapy, we performed a subgroup analysis excluding patients receiving these treatments. This analysis revealed a similar and significant reduction in FPG levels 6 months post-RDN, suggesting that the observed effects on glycaemic control were independent of concurrent antidiabetic medications. The observed correlation between the change in FPG levels and the change in 24-h systolic BP at 3 months suggests that the early metabolic effects of RDN may be closely linked to improvements in BP regulation. However, the precise mechanisms by which RDN affects glucose metabolism remain unclear, although they likely involve complex crosstalk of many factors, including reduced sympathetic outflow through afferent signalling, inhibition of the renin–angiotensin system, improved skeletal muscle blood flow due to decreased α-adrenergic tone and increased glucose uptake [6, 27].

One possible explanation for the greater decrease in FPG in CKD could be the heightened baseline state of SNS overactivity in CKD patients. CKD is associated with SNS overactivity, which contributes not only to hypertension, but also to insulin resistance and impaired glucose metabolism [28]. By disrupting renal sympathetic nerves, RDN can mitigate these effects, potentially leading to improved glucose regulation in CKD patients who are more susceptible to these metabolic disturbances. One can speculate that the observed reduction in glucose levels may be mediated by factors independent of insulin resistance, such as enhanced glucose uptake in peripheral tissues or reduced hepatic glucose production, both of which can be influenced by the SNS [29]. Ott *et al.* [30] demonstrated that RDN enhances pancreatic β cell secretory capacity 6 months post-procedure, which could further explain the improved glucose control observed.

Regarding HbA1c, a significant reduction was noted in the whole study group as well as in those without CKD, after excluding patients with antidiabetic therapy. The lack of significant HbA1c changes in the CKD group may be attributed to the faster turnover of erythrocytes in CKD. Evidence suggests that HbA1c may not reliably reflect glycaemic control in CKD patients. This is primarily due to red blood cell turnover in this population, often resulting from iron deficiency and reduced erythropoietin production due to uraemic toxins, which in turn affects the accuracy of HbA1c as an indicator of long-term glucose levels [31, 32].

In terms of BP, we observed a significant office BP reduction of 13.6/6.7 mmHg and 24-h ABP of 6.1/4.0 mmHg in the whole study population 6 months after RDN. The findings are consistent with those from the Global Symplicity Registry, where a decrease in 24-h ABP of 6.6 mmHg systolic and 3.9 mmHg diastolic was observed 6 months following RDN [33]. Of note, renal function did not significantly change 3 and 6 months after RDN, as previously already reported [34–36].

### Limitations

While these findings are promising, the study has several limitations. The RDN procedure was conducted using heterogeneous devices. So far, the reported data do not support any clinically relevant heterogeneous effect of the technology on BP [37]. Adherence to concomitant medication was not monitored at baseline and after 6 months in all patients. Adherence to antihypertensive medication was measured by urinary toxicological measurements only in a minority of the patients included in the randomized controlled trial using the second-generation catheter system. Future prospective, double-blind and sham-controlled studies are needed to confirm these findings.

## CONCLUSION

We observed a significant and augmented improvement in FPG levels among hypertensive patients with CKD following RDN. Although RDN research has traditionally concentrated on its primary role in reducing BP and managing resistant hypertension, our findings suggest an additional benefit, specifically addressing glucose control. Given that CKD patients often face complex metabolic challenges, including impaired glucose regulation and increased cardiovascular risk, our results may provide new insights into the potential metabolic benefits of RDN for this particularly vulnerable group.

## Data Availability

Data are available upon request to the corresponding author.

## References

[1] Esler M . Sympathetic nervous system: contribution to human hypertension and related cardiovascular diseases. J Cardiovasc Pharmacol 1995;26(Suppl 2): S24–8. 10.1097/00005344-199512020-00004 8642801

[2] Mancia G, Grassi G, Giannattasio C et al. Sympathetic activation in the pathogenesis of hypertension and progression of organ damage. Hypertension 1999;34:724–8. 10.1161/01.HYP.34.4.724 10523349

[3] Malpas SC . Sympathetic nervous system overactivity and its role in the development of cardiovascular disease. Physiol Rev 2010;90:513–57. 10.1152/physrev.00007.2009 20393193

[4] Esler M, Lambert E, Schlaich M. Point: chronic activation of the sympathetic nervous system is the dominant contributor to systemic hypertension. J Appl Physiol 2010;109:1996–8; discussion 2016. 10.1152/japplphysiol.00182.2010 20185633

[5] Esler M . The 2009 Carl Ludwig Lecture: Pathophysiology of the human sympathetic nervous system in cardiovascular diseases: the transition from mechanisms to medical management. J Appl Physiol 2010;108:227–37. 10.1152/japplphysiol.00832.2009 19940096

[6] Carnagarin R, Lambert GW, Kiuchi MG et al. Effects of sympathetic modulation in metabolic disease. Ann NY Acad Sci 2019;1454:80–9. 10.1111/nyas.14217 31424101

[7] Masuo K, Mikami H, Ogihara T et al. Sympathetic nerve hyperactivity precedes hyperinsulinemia and blood pressure elevation in a young, nonobese Japanese population. Am J Hypertens 1997;10:77–83. 10.1016/S0895-7061(96)00303-2 9008251

[8] Scherrer U, Sartori C. Insulin as a vascular and sympathoexcitatory hormone: implications for blood pressure regulation, insulin sensitivity, and cardiovascular morbidity. Circulation 1997;96:4104–13. 10.1161/01.CIR.96.11.4104 9403636

[9] Huggett RJ, Scott EM, Gilbey SG et al. Impact of type 2 diabetes mellitus on sympathetic neural mechanisms in hypertension. Circulation 2003;108:3097–101. 10.1161/01.CIR.0000103123.66264.FE 14676139

[10] Lambert GW, Straznicky NE, Lambert EA et al. Sympathetic nervous activation in obesity and the metabolic syndrome—causes, consequences and therapeutic implications. Pharmacol Ther 2010;126:159–72. 20171982 10.1016/j.pharmthera.2010.02.002

[11] Schlaich MP, Hering D, Sobotka P et al. Effects of renal denervation on sympathetic activation, blood pressure, and glucose metabolism in patients with resistant hypertension. Front Physiol 2012;3:10. 10.3389/fphys.2012.00010 22347190 PMC3270497

[12] Schlaich MP, Sobotka PA, Krum H et al. Renal sympathetic-nerve ablation for uncontrolled hypertension. N Engl J Med 2009;361:932–4. 10.1056/NEJMc0904179 19710497

[13] Kandzari DE, Böhm M, Mahfoud F et al. Effect of renal denervation on blood pressure in the presence of antihypertensive drugs: 6-month efficacy and safety results from the SPYRAL HTN-ON MED proof-of-concept randomised trial. Lancet 2018;391:2346–55. 10.1016/S0140-6736(18)30951-6 29803589

[14] Stavropoulos K, Patoulias D, Imprialos K et al. Efficacy and safety of renal denervation for the management of arterial hypertension: a systematic review and meta-analysis of randomized, sham-controlled, catheter-based trials. J Clin Hypertens (Greenwich) 2020;22:572–84. 10.1111/jch.13827 32049436 PMC8030058

[15] Koutra E, Dimitriadis K, Pyrpyris N et al. Unravelling the effect of renal denervation on glucose homeostasis: more questions than answers? Acta Diabetol 2024;61:267–80. 10.1007/s00592-023-02208-7 38066299 PMC10948574

[16] Mahfoud F, Schlaich M, Kindermann I et al. Effect of renal sympathetic denervation on glucose metabolism in patients with resistant hypertension: a pilot study. Circulation 2011;123:1940–6. 10.1161/CIRCULATIONAHA.110.991869 21518978

[17] Verloop WL, Spiering W, Vink EE et al. Denervation of the renal arteries in metabolic syndrome: the DREAMS-study. Hypertension 2015;65:751–7. 10.1161/HYPERTENSIONAHA.114.04798 25646297

[18] Miroslawska AK, Gjessing PF, Solbu MD et al. Renal denervation for resistant hypertension fails to improve insulin resistance as assessed by hyperinsulinemic-euglycemic step clamp. Diabetes 2016;65:2164–8. 10.2337/db16-0205 27246911

[19] Pourmoghaddas M, Khosravi A, Akhbari M et al. One year follow-up effect of renal sympathetic denervation in patients with resistant hypertension. ARYA Atheroscler 2016;12:109–13. 27429632 PMC4933751

[20] Mancia G, Kreutz R, Brunström M et al. 2023 ESH guidelines for the management of arterial hypertension The Task Force for the management of arterial hypertension of the European Society of Hypertension: endorsed by the International Society of Hypertension (ISH) and the European Renal Association (ERA). J Hypertens 2023;41:1874–2071. 37345492 10.1097/HJH.0000000000003480

[21] Townsend RR, Mahfoud F, Kandzari DE et al. Catheter-based renal denervation in patients with uncontrolled hypertension in the absence of antihypertensive medications (SPYRAL HTN-OFF MED): a randomised, sham-controlled, proof-of-concept trial. Lancet 2017;390:2160–70. 10.1016/S0140-6736(17)32281-X 28859944

[22] Azizi M, Schmieder RE, Mahfoud F et al. Endovascular ultrasound renal denervation to treat hypertension (RADIANCE-HTN SOLO): a multicentre, international, single-blind, randomised, sham-controlled trial. Lancet 2018;391:2335–45. 10.1016/S0140-6736(18)31082-1 29803590

[23] Azizi M, Sanghvi K, Saxena M et al. Ultrasound renal denervation for hypertension resistant to a triple medication pill (RADIANCE-HTN TRIO): a randomised, multicentre, single-blind, sham-controlled trial. Lancet 2021;397:2476–86. 10.1016/S0140-6736(21)00788-1 34010611

[24] Mahfoud F, Weber M, Schmieder RE et al. Catheter-based alcohol-mediated renal denervation for the treatment of uncontrolled hypertension: design of two sham-controlled, randomized, blinded trials in the absence (TARGET BP OFF-MED) and presence (TARGET BP I) of antihypertensive medications. Am Heart J 2021;239:90–9. 10.1016/j.ahj.2021.05.015 34052211

[25] Mahfoud F, Renkin J, Sievert H et al. Alcohol-mediated renal denervation using the Peregrine System Infusion Catheter for treatment of hypertension. JACC Cardiovasc Interv 2020;13:471–84. 10.1016/j.jcin.2019.10.048 32081241

[26] Yakubu-Madus FE, Johnson WT, Zimmerman KM et al. Metabolic and hemodynamic effects of moxonidine in the Zucker diabetic fatty rat model of type 2 diabetes. Diabetes 1999;48:1093–100. 10.2337/diabetes.48.5.1093 10331415

[27] Grassi G . Renal denervation in cardiometabolic disease: concepts, achievements and perspectives. Nutr Metab Cardiovasc Dis 2013;23:77–83. 10.1016/j.numecd.2012.09.004 23149073

[28] Kaur J, Young BE, Fadel PJ. Sympathetic overactivity in chronic kidney disease: consequences and mechanisms. Int J Mol Sci 2017;18:1682. 10.3390/ijms18081682 28767097 PMC5578072

[29] Nonogaki K . New insights into sympathetic regulation of glucose and fat metabolism. Diabetologia 2000;43:533–49. 10.1007/s001250051341 10855527

[30] Ott C, Schmid A, Mahfoud F et al. Secretory capacity of pancreatic beta-cells is enhanced 6 months after renal denervation in hypertensive patients. J Am Coll Cardiol 2018;72:3372–4. 10.1016/j.jacc.2018.09.075 30573036

[31] Vos FE, Schollum JB, Coulter CV et al. Assessment of markers of glycaemic control in diabetic patients with chronic kidney disease using continuous glucose monitoring. Nephrology 2012;17:182–8. 10.1111/j.1440-1797.2011.01517.x 21883672

[32] Copur S, Onal EM, Afsar B et al. Diabetes mellitus in chronic kidney disease: biomarkers beyond HbA1c to estimate glycemic control and diabetes-dependent morbidity and mortality. J Diabetes Complications 2020;34:107707. 10.1016/j.jdiacomp.2020.107707 32861562

[33] Böhm M, Mahfoud F, Ukena C et al. First report of the Global SYMPLICITY Registry on the effect of renal artery denervation in patients with uncontrolled hypertension. Hypertension 2015;65:766–74. 10.1161/HYPERTENSIONAHA.114.05010 25691618

[34] Ott C, Mahfoud F, Mancia G et al. Renal denervation in patients with versus without chronic kidney disease: results from the Global SYMPLICITY Registry with follow-up data of 3 years. Nephrol Dial Transplant 2022;37:304–10. 10.1093/ndt/gfab154 34109413

[35] Kiuchi MG, Graciano ML, Carreira MAM et al. Long-term effects of renal sympathetic denervation on hypertensive patients with mild to moderate chronic kidney disease. J Clin Hypertens 2016;18:190–6. 10.1111/jch.12724 PMC8031511 26718019

[36] Günes-Altan M, Schmid A, Ott C et al. Blood pressure reduction after renal denervation in patients with or without chronic kidney disease. Clin Kidney J 2024;17:sfad237. 10.1093/ckj/sfad237 38186882 PMC10768756

[37] Vukadinović D, Lauder L, Kandzari DE et al. Effects of catheter-based renal denervation in hypertension: a systematic review and meta-analysis. Circulation 2024;150:1599–611. 10.1161/CIRCULATIONAHA.124.069709 39355923 PMC11560572

